# The lipid-lowering drug fenofibrate combined with si-HOTAIR can effectively inhibit the proliferation of gliomas

**DOI:** 10.1186/s12885-021-08417-z

**Published:** 2021-06-03

**Authors:** Wei Zhu, Hongyang Zhao, Fenfen Xu, Bin Huang, Xiaojing Dai, Jikui Sun, Alphonce M. K. Nyalali, Kailiang Zhang, Shilei Ni

**Affiliations:** 1 Department of Neurosurgery, Qilu Hospital, Cheeloo College of Medicine, Shandong University and Institute of Brain and Brain-Inspired Science, Shandong University, Jinan, 250012 Shandong China; 2 Key Laboratory of Brain Function Remodeling, Qilu Hospital of Shandong University, Jinan, 250012 Shandong China; 3 Department of Neurosurgery, Yantai Yuhuangding Hospital, Cheeloo College of Medicine, Shandong University, Yantai, 264000 Shandong China; 4 Department of Pediatrics, Jinan Central Hospital, Cheeloo College of Medicine, Shandong University, Jinan, 250013 Shandong China; 5 The Advanced Technology Genomics Core, The University of Texas MD Anderson Cancer Center, Houston, TX 77030 USA; 6 School of Medicine, Nankai University, 94 Weijin Road, Tianjin, 300071 China; 7 Tianjin Cerebral Vascular and Neural Degenerative Disease Key Laboratory, Tianjin Neurosurgical Institute, Tianjin, 30350 China; 8 Department of Neurosurgery, Tianjin Huanhu Hospital, Tianjin, 300350 China; 9 Department of Orthopedics and Neurosurgery, Mbeya Zonal Referral Hospital and Mbeya University College of Medicine, University of Dar es Salaam, Box 419, Mbeya, Tanzania

**Keywords:** Glioblastoma, Fenofibrate, HOTAIR, PPARA, Therapy

## Abstract

**Background:**

Fenofibrate is a fibric acid derivative known to have a lipid-lowering effect. Although fenofibrate-induced peroxisome proliferator-activated receptor alpha (PPARα) transcription activation has been shown to play an important role in the malignant progression of gliomas, the underlying mechanisms are poorly understood.

**Methods:**

In this study, we analyzed TCGA database and found that there was a significant negative correlation between the long noncoding RNA (lncRNA) HOTAIR and PPARα. Then, we explored the molecular mechanism by which lncRNA HOTAIR regulates PPARα in cell lines in vitro and in a nude mouse glioma model in vivo and explored the effect of the combined application of HOTAIR knockdown and fenofibrate treatment on glioma invasion.

**Results:**

For the first time, it was shown that after knockdown of the expression of HOTAIR in gliomas, the expression of PPARα was significantly upregulated, and the invasion and proliferation ability of gliomas were obviously inhibited. Then, glioma cells were treated with both the PPARα agonist fenofibrate and si-HOTAIR, and the results showed that the proliferation and invasion of glioma cells were significantly inhibited.

**Conclusions:**

Our results suggest that HOTAIR can negatively regulate the expression of PPARα and that the combination of fenofibrate and si-HOTAIR treatment can significantly inhibit the progression of gliomas. This introduces new ideas for the treatment of gliomas.

**Supplementary Information:**

The online version contains supplementary material available at 10.1186/s12885-021-08417-z.

## Background

The incidence of primary brain tumors in the world is approximately 7/100,000 per year. Primary brain tumors account for approximately 2% of primary tumors that occur before the age of 70 and 7% of all cancer deaths. Glioma is the most common primary brain tumor, accounting for approximately 80% of all primary malignant brain tumors, and glioblastoma is the most common type of glioma, accounting for more than 50% of all brain glial tumors, and is the most aggressive among all gliomas (WHO grade IV) [1]. Glioblastoma (GBM) is characterized by uncontrolled cell proliferation, diffuse infiltration, tendency for necrosis, strong angiogenesis, strong resistance to apoptosis, and genomic instability. As reflected in the old name “multiforme”, GBM exhibits significant intratumoral heterogeneity at the cytopathological, transcriptional and genomic levels [1]. These characteristics make GBM one of the most difficult malignancies to analyze and treat. Despite the implementation of intensive treatment strategies and supportive care, the median survival of GBM has remained at 12 months during the past decade [2]. Therefore, continued in-depth study of the pathogenesis of GBM and the development of new targeted therapy methods based on its histomorphological, molecular and genomic characteristics remain crucial scientific research topics in the field of neurosurgical oncology.

In recent years, noncoding RNAs (ncRNAs) have been shown to be involved in the malignant progression of tumors and to play an important role in tumor proliferation, cell cycle progression, angiogenesis, apoptosis and invasion [3, 4]. This type of regulatory RNA that does not encode proteins mainly includes microRNAs, lncRNAs, nucleolar small RNAs, and interfering RNAs (siRNAs). Among these RNAs, miRNAs and lncRNAs have been the most studied. miRNAs regulate gene expression at the posttranscriptional level by targeting the 3′-untranslated region (3′ UTR) of specific messenger RNAs (mRNAs). lncRNAs have been shown to be related to chromosome modification, transcription and posttranscriptional regulation [5–7].

Peroxisome proliferator-activated receptors (PPARs) are members of the nuclear receptor superfamily involved in fatty acid oxidation and glucose and lipid metabolism. They are composed of three subtypes: PPARα, PPARβ, and PPARγ. PPARα participates in lipid metabolism and plays an important role in regulating cell growth, differentiation, and apoptosis. Recent research results suggest that PPARα is involved in the malignant progression of tumors [8]. Fenofibrate, as a PPARα agonist, can inhibit the growth of gliomas in animal models of gliomas [9]. In addition, experimental results show that noncoding RNAs are involved in the regulation of the PPARα signaling pathway [10]. However, the specific control mechanism remains to be further explored.

HOTAIR (Hox transcript antisense intergenic RNA) is a ~ 2.2-kb-long noncoding RNA transcribed from the HOXC locus [11]. Our past research confirmed that HOTAIR promotes glioblastoma cell cycle progression in an EZH2-dependent manner [12]. This study was intended to explore the mechanism of lncRNA HOTAIR in regulating PPARα, to provide an in-depth description of the role of HOTAIR in the malignant progression of glioma by in vivo animal experiments and in vitro cell line experiments and to verify the therapeutic effect of the combination of si-HOTAIR and the PPARα agonist fenofibrate on glioma cells. This study provides an experimental basis for us to understand the pathogenesis of GBM and new ideas for the molecular treatment of gliomas in clinical settings.

## Methods

### Patient samples

A total of 702 glioma samples from TCGA (The Cancer Genome Atlas), including 530 low-grade glioma and 172 glioblastoma samples, were used in this study. The data related to the changes in the binding of EZH2 and H3K27me3 proteins in the PPARΑ promoter region in glioma samples of different grades were obtained from the GEO database (GSE126396, the genome-wide distribution of EZH2 and H3K27me3 in cells at different stages of de novo neoplastic transformation was characterised by ChIP-seq, in duplicate, using an Illumina HiSeq 2500). Software IGV 2.8.6 was used for analysising database GSE126396. Glioma tissues from patients for histopathological staining were derived directly from glioma patients who underwent surgery at Qilu Hospital.)

### Cell culture

The human GBM cell lines U87 and U251 were purchased from ATCC (Manassas, Virginia, USA) in August 2015. After obtaining the U87 and U251 cell lines, they were immediately cultured for proliferation and frozen in liquid nitrogen for subsequent studies. U251 cells were cultured in complete MEM (Gibco, Invitrogen, Paisley, UK) medium, while U87 cells were cultured in DMEM (Gibco, Invitrogen, Paisley, UK) medium containing 10% FBS (fetal bovine serum) at 37 °C in 5% CO_2_. With the exception of the in vivo cultures, all glioblastoma cells were maintained for less than eight generations.

### GO analysis, survival curve plot, correlation plot and heat map

GO analysis was performed using the Cluster Profiler [13] R package. A survival curve plot was generated using the survminer (https://github.com/kassambara/survminer/issues) R package. A correlation plot was generated using the corrplot (https://github.com/taiyun/corrplot) R package. Heat map plots were built using cluster 3.0 (Stanford University).

### Western blot analysis

Protein was extracted from cells that were cultured without FBS and treated with drugs following a concentration gradient for 24 h. Equal amounts of total protein (nucleoprotein) per lane were separated using 10% or 15% SDS-polyacrylamide gel electrophoresis and then transferred to a PVDF membrane. The membrane was blocked in 5% skim milk for 1 h and then incubated with primary antibody for 2 h. The primary antibodies used were anti-H3K27me3 (Cell Signaling Technology, USA), anti-PPARα (Proteintech, Rosemont, IL) and anti-GAPDH (Proteintech, Rosemont, IL). Detection of protein bands was performed using a Super Signal protein detection kit (Pierce, USA). The band densities of specific proteins were quantified after normalization to the density of the GAPDH band.

### Analysis of wound healing and perforation migration

U87 and U251 cells were infected with control or lentivirus containing the si-HOTAIR segment, after which the cells were treated with the presence or absence of 100 μM fenofibrate (Sigma-Aldrich, Germany). Cells in each group (control, si-HOTAIR, si-HOTAIR+fenofibrate) were seeded into 6-well plates. HOTAIR siRNA segments (the HOTAIR siRNA sequence was 5′-GAACGGGAGUACAGAGAGAUU-3′) were obtained from GenePharma (Shanghai, China). After 24 h, a 200 μl sterile pipette tip was used to evenly scrape a single layer of cells. The cell debris was removed by washing with PBS (phosphate buffered saline). Then, the cells were incubated with serum-free medium under normal conditions. Photos were taken at 0 and 48 h after scratching. The wound area was evaluated by ImageJ, and the percentage of wound healing was calculated as follows: (0 h wound area-48 h wound area) / 0 h wound area × 100.

For the in vitro migration assays, U87-control, U87-si-HOTAIR, U87-si-HOTAIR+fenofibrate, U251-control, U251-si-HOTAIR, and U251- si-HOTAIR+fenofibrate cells were seeded into the upper culture chamber of a 24-well Transwell plate. The medium in the upper chamber contained no serum, and the medium in the lower chamber contained 10% FBS. After incubation at 37 °C for 48 h, nonmigrating cells on the upper surface of the membrane were removed with a cotton swab. Cells that passed through the membrane were fixed with methanol, stained with crystal violet, and counted in 5 random fields under an optical microscope (100x magnification).

### Colony formation test

After treating U87 and U251 cells with control, si-HOTAIR or si-HOTAIR+fenofibrate, the cells were inoculated into 6-well plates and incubated. After 14 days, the cells were fixed with 4% paraformaldehyde and stained with 0.1% crystal violet. We used a digital camera to capture pictures from different transfected colonies (more than 50 cells per clone).

### Chromatin immunoprecipitation (ChIP) and ChIP-qPCR assays

ChIP assays were performed using the commercially available ChIP Assay Kit (Beyotime). The ChIP-qPCR primers used were as follows: forward-1: AACCTTGGGAGCCCCAAAAA and reverse-1: GTGCAGAGTGGTCACGTACA; forward-2: GAGCGTGGTTTCCCAGAAGA and reverse-2: TTTGGGGCTCCCAAGGTTTT; forward-3: TACGTGACCACTCTGCACAC and reverse-3: CCTCCGGGCTCAAAGACATT; forward-4: TACGTGACCACTCTGCACAC and reverse-4: CCTCCGGGCTCAAAGACATT.

### Nude mouse intracranial glioma model and in vivo bioluminescence imaging

BALB/c-A nude mice were purchased from the Animal Center of Cancer Institute of Chinese Academy of Medical Sciences (Beijing), and their care was carried out in accordance with institutional guidelines. Three- to five-week-old mice were selected as the intracranial tumor model. U87 and U87-si-HOTAIR cells (5 × 10^5^) were stereotactically injected into the right striatum of nude mice. Seven days after injection, 200 mg/kg fenofibrate suspended in 5% sodium carboxymethylcellulose was given daily via intragastric administration in the treatment group, while an equal volume of 5% sodium carboxymethylcellulose was administered in the control group. The treatment lasted 21 days. The growth of intracranial tumors was monitored on days 7, 14, 21, and 28 using a bioluminescence imaging system.

### Histopathological staining

The whole brains of the mice were harvested on day 28 after the allogeneic glioma cells were implanted, fixed in 4% paraformaldehyde, embedded in paraffin, and cut into 15 μm thick coronal slices. The sections with the largest tumor area were stained with hematoxylin and eosin or were used for IHC staining.

For IHC analysis, sections were incubated with primary antibodies [1:100 dilution; anti-PPRA antibodies were purchased from Cell Signaling Technology (Proteintech), the anti-Ki67 antibody was purchased from Zsgb Bio (Beijing, China) and the anti-CD34 antibody was purchased from Abcam] overnight at 4 °C, followed by a 1 h incubation at 37 °C with a biotinylated secondary antibody (1:100 dilution). The samples were then incubated with horseradish peroxidase-labeled streptomycoidin and DAB (diaminobenzidine), counterstained with hematoxylin, and visualized using a light microscope.

For the IHC analysis, we quantitatively scored the tissue sections according to the percentage of positive cells and staining intensity. We assigned the following proportion scores: 1 if 0–25% of the tumor cells showed positive staining, 2 if 26–50% of cells were stained, 3 if 51–75% of the cells were stained, and 4 if 76–100% of the cells were stained; we also divided the different expression levels into four different groups (1 to 4) and scored them.

### Statistical analysis

Statistical analyses were performed using GraphPad Prism 6.0 (GraphPad, La Jolla, CA, USA). The data are expressed as the mean ± SD and analyzed by one-way analysis of variance for multiple comparisons or Student’s t-test (two-tailed) for comparing two groups. Statistical significance was set at a value of *P* < 0.05.

## Results

### The expression of HOTAIR and PPARα in glioma showed a negative correlation

**Fig. 1:**
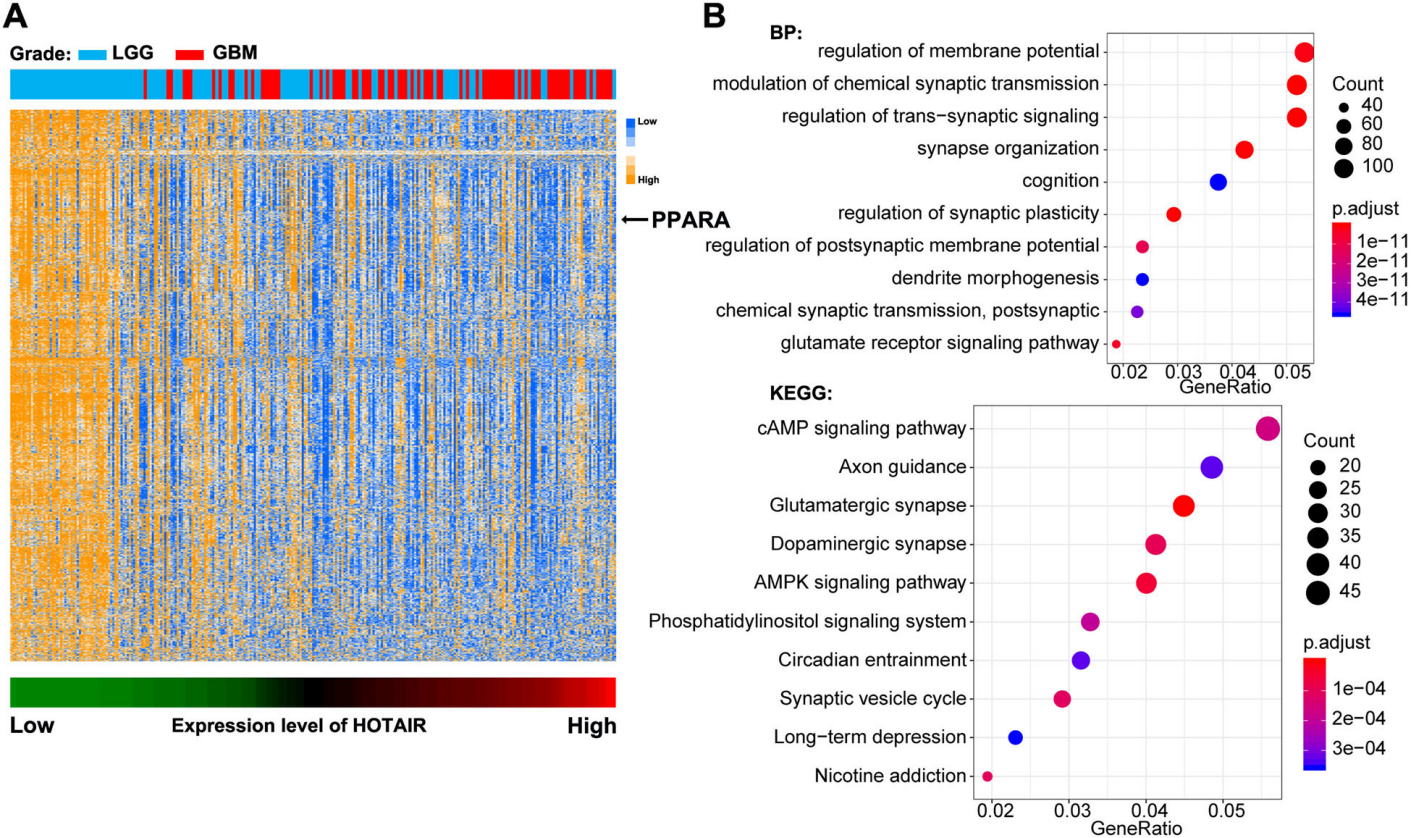
HOTAIR and PPARα expression in glioma were negatively correlated. **A** Expression heat map of genes in the TCGA glioma database that were significantly negatively related to HOTAIR expression. The genes are arranged according to the changes in HOTAIR expression. **B** GO analysis and KEGG analysis of genes negatively related to HOTAIR expression

To verify the regulatory mechanism of the HOTAIR gene on the occurrence and progression of glioma, we heat-mapped approximately 2600 genes negatively related to HOTAIR expression. The results suggest that almost all genes were highly expressed in low-grade gliomas (Fig. 1A) and that the expression level decreased as the tumor grade increased. Further GO analysis of these genes showed that their biological processes were regulation of membrane potential, modulation of chemical synaptic transmission and regulation of transsynaptic signaling and synapse organization (Fig. 1B).

We then analyzed the signaling pathways enriched in these genes and found mainly the cAMP signaling pathway, AMPK signaling pathway and phosphatidylinositol signaling system (Fig. 1B). Most of these signaling pathways have been confirmed by scholars to be involved in the progression of tumors and different degrees of abnormal cell behaviors [14]. Excessive activation of phosphatidylinositol can lead to abnormal cell proliferation, abnormal endocytosis and exocytosis, abnormal cell metastasis and even tumor development [15]. AMP kinase promotes the bioenergy and tumor growth of glioblastomas [16].

**Fig. 2:**
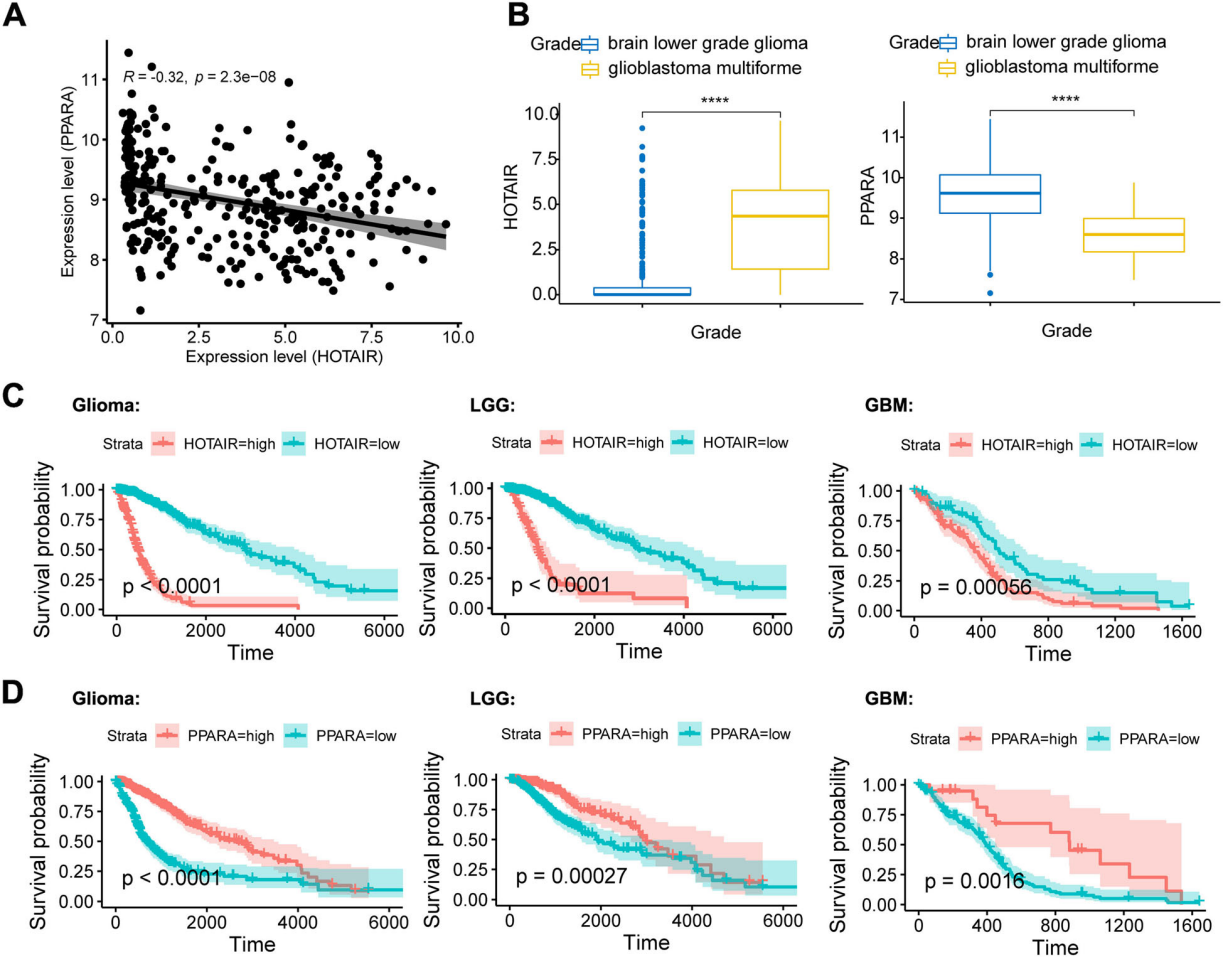
HOTAIR and PPARα had a significant negative correlation. The low expression of PPARα and high expression of HOTAIR predicted a poor prognosis. **A** Scatter plots of HOTAIR and PPARα expression. r, Pearson product-moment correlation coefficient. *p, p* value. **B** Box diagram of HOTAIR and PPARα expression in different grades of glioma. ****, *p* < 0.0005. **C** Survival curve plots between different HOTAIR expression groups. **D** Survival curve plots between different PPARα expression groups

The AMPK pathway and PPARA are both involved in metabolic activities such as fatty acid oxidation, blockade of glycolysis and protein, nucleotide and fatty acid synthesis. We further examined the expression of HOTAIR and PPARα in gliomas. The results suggest that there was a significant negative correlation between them. The Pearson correlation coefficient was − 0.32 (Fig. 2A). By comparing the expression levels of HOTAIR and PPARα in low-grade gliomas and glioblastomas, it was found that the expression of HOTAIR in GBM was upregulated, while PPARα was expressed at low levels (Fig. 2B). In the results of the survival analysis, it was found that the high expression of HOTAIR indicated a poor prognosis, but the high expression of PPARΑ indicated a good survival prognosis (Fig. 2C and D). These results indicated that there is a negative correlation between the expression of lncRNA HOTAIR and PPARα in gliomas and that it has significance in indicating the survival rate and clinical grade.

### PPARΑ expression decreased in clinical samples of patients with high-grade glioma

**Fig. 3:**
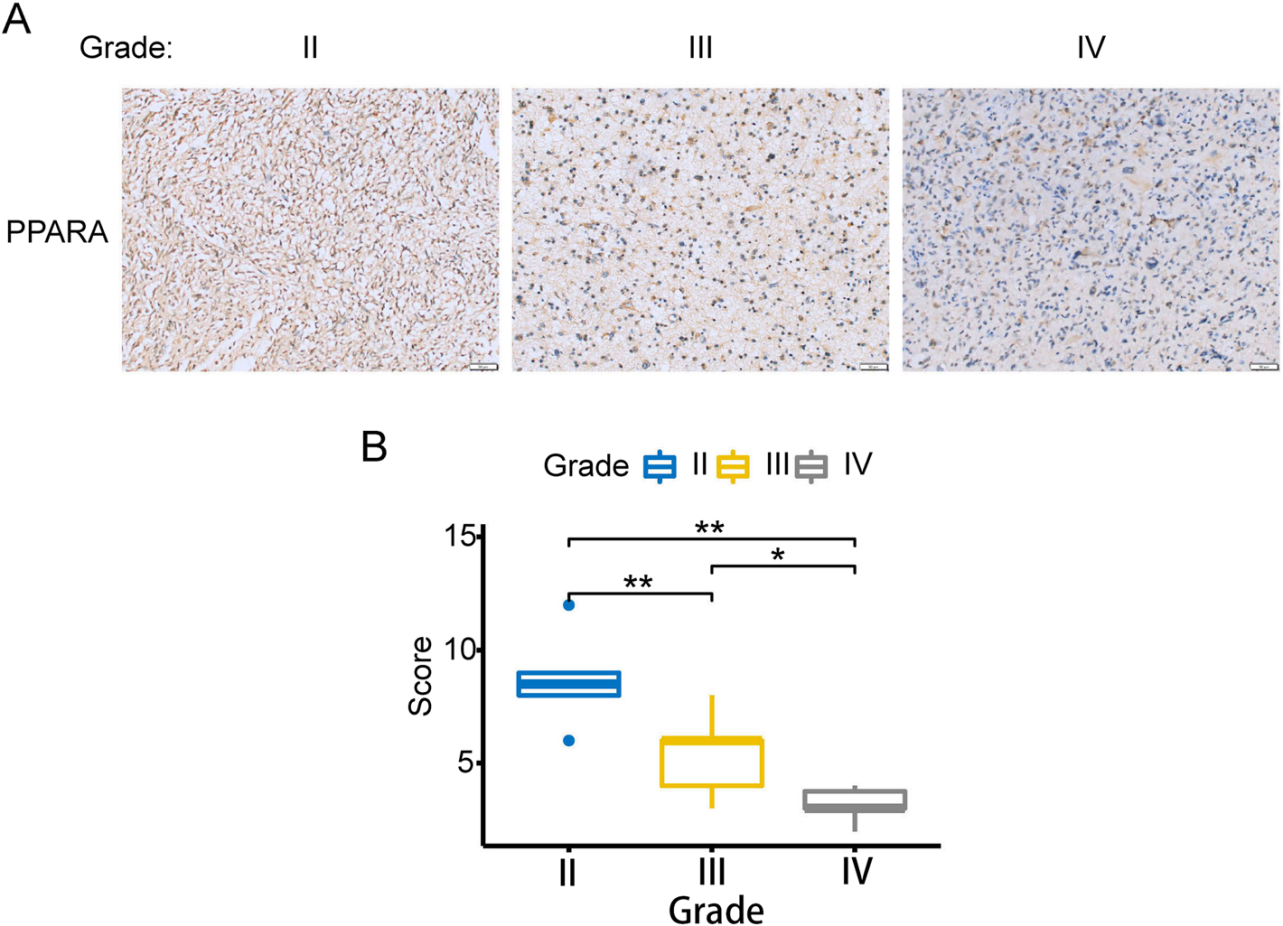
PPARα expression decreased in clinical samples of patients with high-grade glioma. **A** PPARα immunohistochemical staining of glioma samples of different grades. **B** Statistical results of the immunohistochemical staining. The data are shown as the mean ± SD. *, *P* < 0.05; **, *P* < 0.01

Through survival analysis of GBM (WHO grade IV), we found that the low expression level of PPARΑ in the TCGA database was significantly associated with a poor prognosis for patients (Fig. 2D). To avoid bias from the analysis of only mRNA expression data, we examined the PPARΑ protein levels of 21 different grades of glioma samples by immunohistochemical (IHC) staining (the clinical information is listed in Supplementary Table 1). As shown in Fig. 3A, we observed a significant decrease in PPARΑ protein levels in high-grade gliomas. Quantification of the staining (see Methods) revealed a strong association between the abundance of PPARΑ-positive cells and lower tumor grade (Fig. 3B). Therefore, the downregulated expression level of PPARΑ is an indicator of the aggressiveness of malignant gliomas.

### The expression level of HOTAIR enhanced the degree of H3K27me3 binding to the PPARΑ promoter

**Fig. 4:**
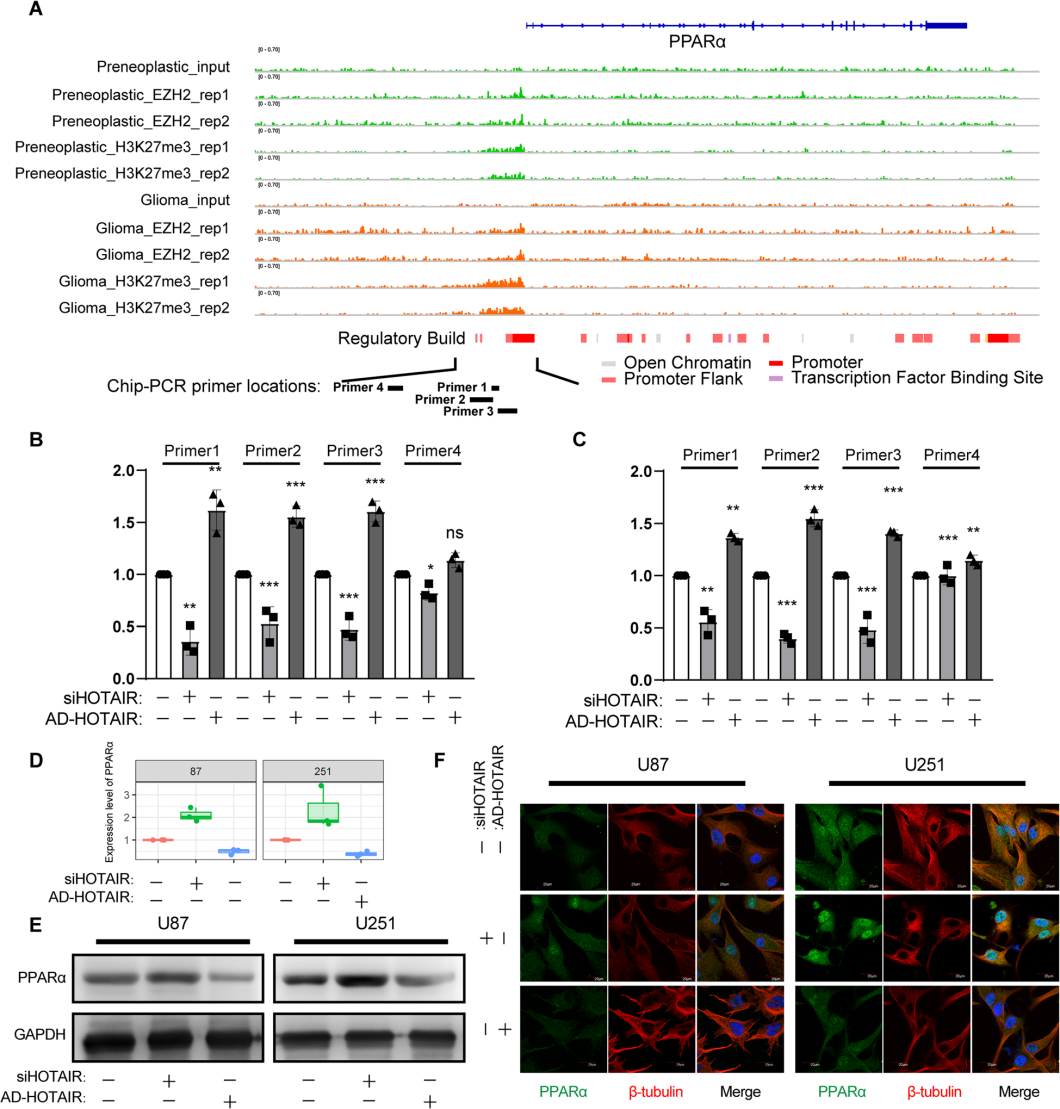
HOTAIR enhanced the degree of H3K27me3 binding to the PPARα promoter. **A** EZH2 and H3K27me3 proteins bind to the promoter region of the PPARα gene in different grades of glioma. **B** ChIP analysis of the H3K27me3 protein in the promoter region of the PPARα gene in different treatment groups in the U87 cell line. **C** ChIP analysis of the H3K27me3 protein in the promoter region of the PPARα gene in different treatment groups in the U251 cell line. **D** Quantitative PCR analysis. **E** Western blot analysis; full-length blots are presented in Supplementary Fig. S1. F) Immunofluorescence analysis. Bar, 20 μm. The data are shown as the mean ± SD. *, *P* < 0.05; **, *P* < 0.01

H3K27me3 is a histone modification related to transcriptional repression. HOTAIR can promote the formation of H3K27me3 through the EZH2 protein, silence the transcriptional expression of genes and promote the malignant progression of glioma. Analysis of the GSE126396 database showed that there was a significant difference in the amount of H3K27me3 protein enrichment in the PPARΑ promoter region of glioma samples of different malignancies (Fig. 4A). To investigate whether HOTAIR overexpression mediated PPARΑ promoter gene silencing, we analyzed the binding of HEK27me3 in the PPARΑ promoter region in control, HOTAIR and si-HOTAIR GBM cell lines by ChIP-PCR. The experimental results showed that the high expression of HOTAIR promoted the enrichment of H3K27me3 protein in the PPARΑ promoter region, while reducing the expression of HOTAIR in glioma cell lines reduced the amount of binding (Fig. 4B and C). RT-PCR, western blot and immunofluorescence experiments were used to analyze the expression of PPARΑ mRNA and protein in control, HOTAIR and si-HOTAIR GBM cell lines. The expression of HOTAIR had a negative regulatory relationship with PPARΑ (Fig. 4D~F) (Full-length blot showed in Fig. S1). Based on the above findings, we concluded that the EZH2-H3K27me3 pathway mediated by HOTAIR can silence the transcriptional expression of PPARΑ.

### Low expression of HOTAIR and activation of the PPARΑ pathway weakened the migration and colony-forming ability of U87 and U251 glioma cells

**Fig. 5:**
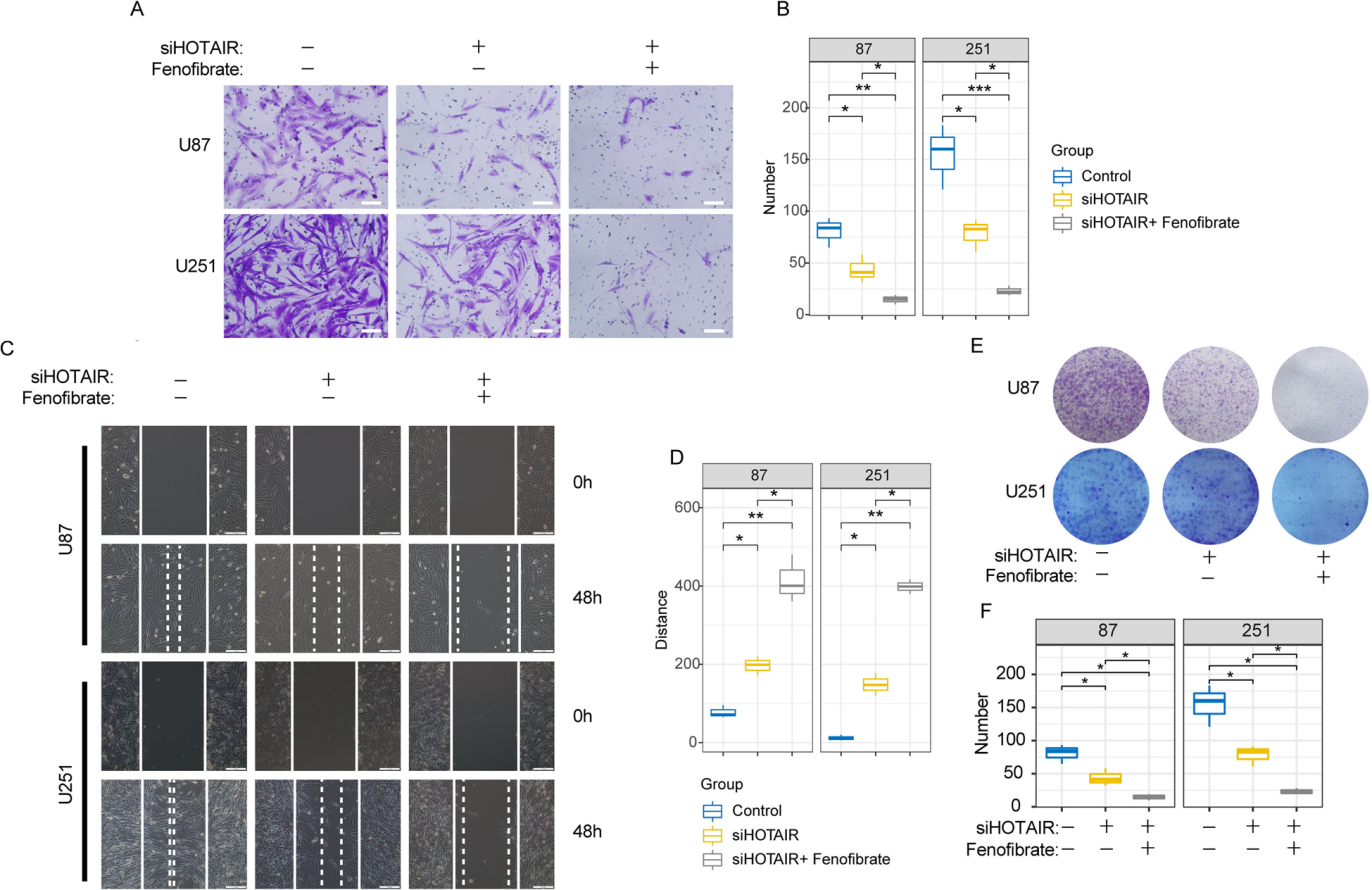
si-HOTAIR and fenofibrate combined therapy attenuated the migration and colony formation abilities of U87 and U251 glioma cells. **A** Transwell analysis. White bar, 200 μm. **B** Statistical analysis of the transwell assay. The data are shown as the mean ± SD. *, *P* < 0.05; **, *P* < 0.01. **C** Wound-healing assay. White bar, 200 μm. **D** Statistical analysis of the wound-healing assay. The data are shown as the mean ± SD. *, *P* < 0.05; **, *P* < 0.01. **E** Colony formation assay. **F** Statistical analysis of the colony formation assay. The data are shown as the mean ± SD. *, *P* < 0.05; **, *P* < 0.01

To study the role of HOTAIR in gliomas, we transfected U87 and U251 glioma cells with si-HOTAIR lentivirus. Transwell migration, wound healing and colony formation analyses were performed to examine the effect of HOTAIR on glioma cell migration and colony formation. As shown by our results, U87 and U251 cells with low expression of HOTAIR showed lower migration ability than the control cells (Fig. 5A, C and E). The statistical results also showed the same results (Fig. 5B and D). In further experiments, we treated si-HOTAIR group glioma cell lines with fenofibrate (100 μM) and found that the cell migration and colony-formation ability were further decreased. These results indicated that the activation of the PPARΑ pathway can suppress the malignant phenotype of gliomas and that the combination of downregulation of HOTAIR and activation of the PPARΑ pathway can more effectively inhibit the malignant progression of gliomas.

### Reducing HOTAIR expression in glioma cells combined with fenofibrate treatment effectively inhibited the growth of glioma xenografts

**Fig. 6:**
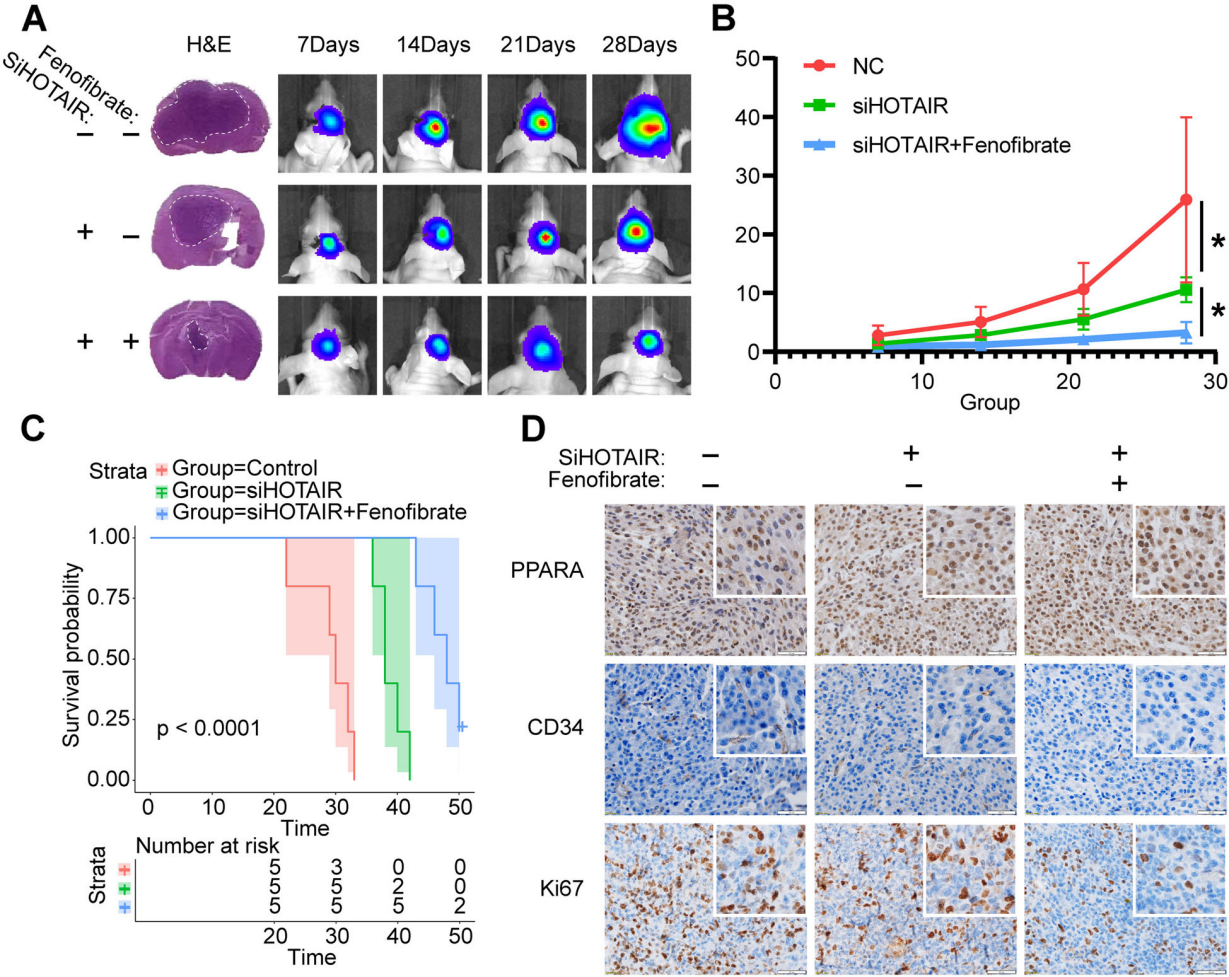
si-HOTAIR and fenofibrate combined therapy slowed glioma growth. **A** H&E staining of different groups of U87 intracranial glioma models. Tumor volume was measured every 7 days by in vivo imaging. **B** Statistical analysis results of the control and combined therapy intracranial glioma groups for every 7 days. The data are shown as the mean ± SD. *, *P* < 0.05. **C** Survival curve plots of the control and combined therapy intracranial glioma groups. **D** Immunohistochemical staining. White bar, 50 μm

To investigate whether reducing HOTAIR expression combined with fenofibrate treatment will reduce the growth of glioma in vivo, nude mice were implanted intracranially with U87 cells infected with a lentivirus containing a si-HOTAIR segment or a control lentivirus to form glioma xenografts. The si-HOTAIR group was randomly divided into control treatment and fenofibrate treatment groups. Seven days after injection, 200 mg/kg fenofibrate suspended in 5% sodium carboxymethylcellulose was given daily via intragastric administration in the treatment group, while an equal volume of 5% sodium carboxymethylcellulose was administered in the control group. The treatment lasted 21 days. On days 7, 14, 21, and 28 after implantation, bioluminescence imaging of the NC, si-HOTAIR and combination therapy groups showed that the BLI signal intensity of the combination therapy group was significantly lower than that of the NC and si-HOTAIR groups (Fig. 6A and B). For explaining the necessity of combination medication, a separate trial was conducted between the control group using fenofibrate alone and combination group in vivo (Fig. S2). Our results suggest that combination therapy provides better treatment results than fenofibrate alone. The HE staining results were consistent with the bioimaging results (Fig. 6A). Survival analysis showed that the prognosis of the mice in the combined treatment group was significantly better (Fig. 6C). Further immunohistochemical staining showed that after reducing the HOTAIR expression of gliomas, the expression of PPARΑ protein was increased, and the CD34 and Ki67 protein expression of the combined treatment group was lower than that of the other two groups. These results indicated that in an in vivo environment, reducing the expression of HOTAIR combined with treatment with fenofibrate can effectively inhibit tumor proliferation and growth.

## Discussion

GBM is the most common and deadly cancer of the central nervous system [17, 18]. However, current conventional treatments, such as surgical resection, radiotherapy and chemotherapy, do not significantly improve the patient’s prognosis. Therefore, there is an urgent need to further study the molecular pathways of glioma development and explore feasible therapeutic options. Fenofibrate is an effective ligand for peroxisome proliferator-activated receptor alpha (PPARα) and has historically been used to regulate glucose and lipid metabolism in the treatment of different forms of hyperlipidemia and hypercholesterolemia [19]. In recent years, fenofibrate has proven to have interesting anticancer effects [20–24].

HOTAIR regulates the expression of a variety of proteins through PRC2 [6, 25]. Studies by other scholars have confirmed that HOTAIR expression levels in tumors are higher than those in corresponding noncancerous tissues. Its high expression predicts a poor prognosis [6, 25, 26]. In our previous studies, HOTAIR was considered not only an important marker of tumor classification and prognosis but also an important marker of glioma molecular subtypes [5, 27, 28].

In this study, we analyzed the expression of HOTAIR based on the RNA expression database of 702 TGGA glioma patients, analyzed the genes negatively related to HOTAIR expression using bioinformatics analyses and found that many of these genes were involved in tumor formation (Fig. 1A and B). Correlation analysis revealed that there was a significant negative correlation between HOTAIR and PPARα (Fig. 2A), and low expression of PPARα was shown through survival analysis to predict a poor prognosis (Fig. 2D). Through immunohistochemical staining of clinical samples, it was shown that with increasing glioma grade, the expression level of PPARα decreased significantly (Fig. 3). Therefore, we predicted that HOTAIR can reduce the expression of PPARα through the PRC2 protein, thereby promoting the malignant progression of glioma.

Through ChIP-PCR analysis, it was shown that after knockdown of the expression of HOTAIR in glioblastoma, the binding of histone H3K27me3 in the promoter region of the PPARα gene was significantly reduced (Fig. 4A). Through quantitative PCR, western blot and immunofluorescence, it was found that the expression of PPARα increased significantly with the decrease in HOTAIR expression (Fig. 4B ~ F). These results indicated that HOTAIR can increase the amount of H3K27me3 binding in the PPARα promoter region through PRC2, thereby silencing the transcriptional expression of PPARα. In further in vitro migration experiments, colony formation experiments and scratch experiments, it was found that after the combination of si-HOTAIR and fenofibrate treatment, the migration and colony-formation ability of glioma cells decreased significantly (Fig. 5). The in vivo malignant glioma model also showed that combined therapy can significantly reduce the rate of tumor cell proliferation, thereby extending the survival period of nude mice (Fig. 6).

## Conclusions

Our study not only explores the molecular mechanism by which HOTAIR regulates PPARα but also verifies the therapeutic effect of the combined application of the PPARα agonist fenofibrate and siRNA HOTAIR on glioma through in vivo animal experiments. This provides new ideas for the comprehensive treatment of gliomas.

## Supplementary Information

**Additional file 1: Table S1.** Information of 21 glioma samples with different grades. **Figure S1.** Full-length blots for blot figures in Fig. 4E. **Figure S2.** Fenofibrate and si-HOTAIR combined therapy slowed glioma growth. (A) Tumor volume was measured every seven days by *in vivo* imaging. (B) Survival curve plots of the control and combined therapy intracranial glioma groups. (C) Tumor growth curves were evaluated. The data are shown as the mean ± SD. *, *P* < 0.05.

## Data Availability

The datasets used and analyzed during the current study are available from the corresponding author upon reasonable request.

## Abbreviations

HOTAIR: HOX transcript antisense RNA
PPARα: Peroxisome proliferator-activated receptor alpha
lncRNA: Long noncoding RNA
GBM: Glioblastoma
3′ UTR: 3′-untranslated region
TCGA: The Cancer Genome Atlas
ChIP: Chromatin immunoprecipitation
IHC: Immunohistochemical
PCR: Polymerase chain reaction
PRC2: Polycomb repressive complex 2
EZH2: Enhancer of zeste homolog 2
AMPK: Adenosine monophosphate-activated protein kinase
PVDF: Polyvinylidene fluoride
