# Supplementary material for: The lipid-lowering drug fenofibrate combined with si-HOTAIR can effectively inhibit the proliferation of gliomas
Source: BMC Cancer. 2021 Jun 3;21:664. doi: 10.1186/s12885-021-08417-z (PMC8173837; doi:10.1186/s12885-021-08417-z)

**The lipid-lowering drug fenofibrate combined with si-HOTAIR can effectively inhibit the proliferation of gliomas**

Wei Zhu^1,2,3^, Hongyang Zhao^4^, Fenfen Xu^4^, Bin Huang^1,2^, Xiaojing Dai^5^, Jikui Sun^6,7^, Alphonce M.K Nyalali^1,2,8^ , Kailiang Zhang^1,2^*, Shilei Ni^1,2^*

*Correspondence: Kailiang-zhang@email.sdu.edu.cn (K. Zhang); [ytfishn@126.com](mailto:ytfishn@126.com) (S. Ni).

^1^Department of Neurosurgery, Qilu Hospital, Cheeloo College of Medicine, Shandong University and Institute of Brain and Brain-Inspired Science, Shandong University, Jinan 250012, Shandong, China;

Full list of author information is available at the end of the article.

**Supplementary Table 1. Information of 21 glioma samples with different grades.**

| Grade | Age | Gender | Score |
| --- | --- | --- | --- |
| IV | 63 | female | 2 |
| IV | 55 | male | 4 |
| IV | 61 | female | 3 |
| IV | 46 | male | 3 |
| IV | 70 | female | 3 |
| IV | 61 | male | 4 |
| III | 51 | female | 6 |
| III | 45 | male | 6 |
| III | 35 | female | 3 |
| III | 45 | female | 8 |
| III | 72 | male | 6 |
| III | 55 | male | 3 |
| III | 39 | female | 4 |
| III | 47 | male | 6 |
| III | 57 | female | 4 |
| II | 43 | male | 6 |
| II | 49 | male | 9 |
| II | 52 | female | 9 |
| II | 50 | male | 8 |
| II | 39 | female | 8 |
| II | 33 | female | 12 |

**Supplementary Figure S1. Full-length blots for blot figures in figure 4E.**


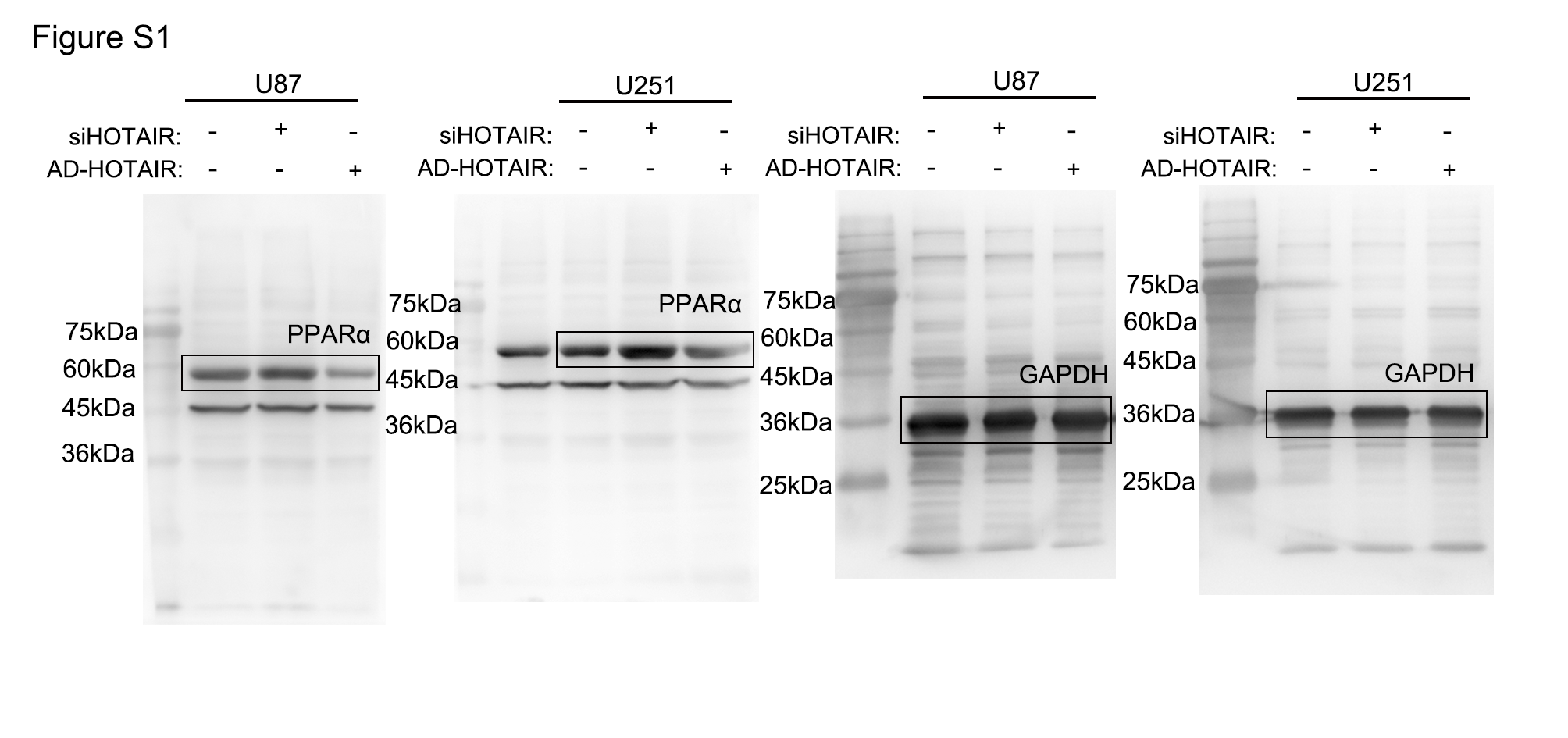


**Supplementary Figure S2.** Fenofibrate and si-HOTAIR combined therapy slowed glioma growth. (A) Tumor volume was measured every seven days by *in vivo* imaging. (B) Survival curve plots of the control and combined therapy intracranial glioma groups. (C) Tumor growth curves were evaluated. The data are shown as the mean ± SD. *, *P* < 0.05.


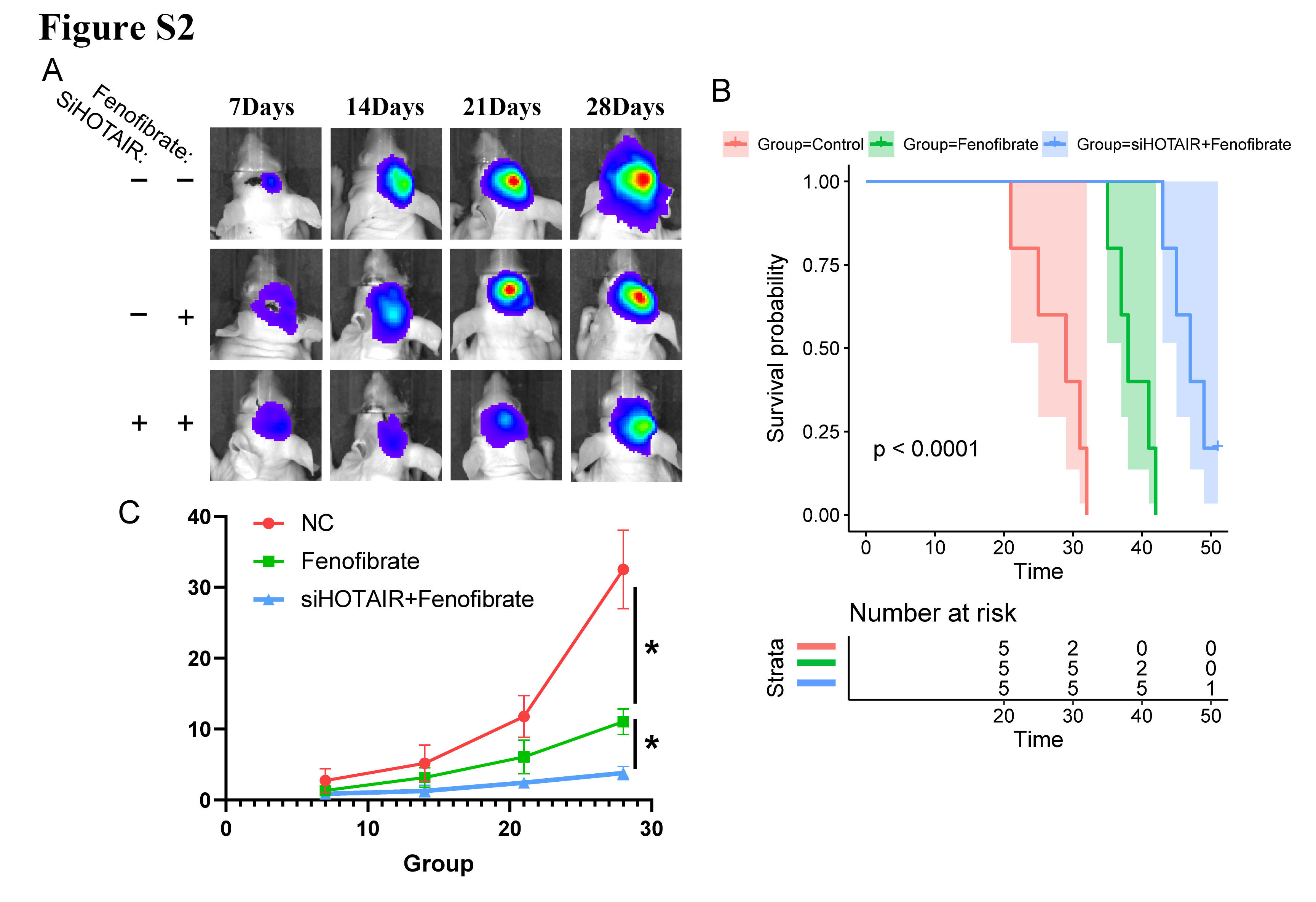

Supplement: Supplementary file 1 — Additional file 1: Table S1. Information of 21 glioma samples with different grades. Figure S1. Full-length blots for blot figures in Fig. 4E. Figure S2. Fenofibrate and si-HOTAIR combined therapy slowed glioma growth. (A) Tumor volume was measured every seven days by in vivo imaging. (B) Survival curve plots of the control and combined therapy intracranial glioma groups. (C) Tumor growth curves were evaluated. The data are shown as the mean ± SD. *, P < 0.05. [file 12885_2021_8417_MOESM1_ESM.docx]
